# VIRTUAL LEARNING ENVIRONMENT IN PEDIATRIC RHEUMATOLOGY FOR PEDIATRIC RESIDENTS

**DOI:** 10.1590/1984-0462/2020/38/2018189

**Published:** 2020-01-13

**Authors:** Ana Luiza Garcia Cunha, Maria Teresa Terreri, Claudio Arnaldo Len

**Affiliations:** a Fundação Hospitalar do Estado de Minas Gerais, Belo Horizonte, MG, Brazil.; b Universidade Federal de São Paulo, São Paulo, SP, Brazil.

**Keywords:** Rheumatology, Teaching, Education, medical, Medical informatics, Education, distance, Pediatrics, Reumatologia, Ensino, Educação médica, Informática médica, Educação a distância, Pediatria

## Abstract

**Objective:**

To develop, implement and evaluate an online virtual learning environment (VLE) on pediatric rheumatology, aimed at pediatric residents, analyzing its effectiveness and satisfaction rates.

**Methods:**

A total of 92 first and second year pediatric residents at two pediatric reference centers were invited to participate in the study. Residents were randomized into a case group (that answered the pre-course test, attended the six virtual pediatric rheumatology modules, and then responded to the post-course test and a satisfaction questionnaire) and a control group (that only answered the pre-course test and, after 4 weeks, the post-course test).

**Results:**

Forty-seven residents (51%) completed their participation. In the case group (n=24), the mean percentage of correct answers was 14% higher on the post-course test (p<0.001). The number of correct answers was larger in the case group than in the control one (n=23) in the post-course test (p=0.045). In the assessment of satisfaction with VLE use, residents considered the site easy to navigate (91%), suitable as a learning tool (91%), and attractive in design (79%). They reported poor prior knowledge in pediatric rheumatology (91%) and agreed that there was good learning with the methodology (75%).

**Conclusions:**

The virtual learning environment in pediatric rheumatology proved to be an effective teaching tool with high satisfaction rates, providing pediatrician residents with adequate knowledge regarding the initial assessment and management of children with rheumatic diseases.

## RESUMO

**Objetivo:**

Elaborar, implementar e avaliar um ambiente virtual de aprendizagem ***online*** em reumatologia pediátrica, direcionado aos residentes em pediatria, analisando sua efetividade e seus índices de satisfação.

**Métodos:**

Foram convidados 92 residentes de pediatria do primeiro e segundo anos de dois centros de referência em pediatria. Os residentes foram divididos, de forma randomizada, em grupo caso (que respondeu ao teste pré-curso, assistiu aos seis módulos virtuais de reumatologia pediátrica e, ao término das aulas virtuais, respondeu ao teste pós-curso e ao questionário de satisfação) e grupo controle (que apenas respondeu ao teste pré-curso e, após quatro semanas, ao teste pós-curso).

**Resultados:**

Completaram a participação 47 (51%) residentes. No grupo caso (n=24), o percentual de acertos foi 14% maior no teste pós-curso (p<0,001). Houve um percentual de acertos maior no grupo caso em relação ao grupo controle (n=23) na comparação do resultado do teste pós-curso (p=0,045). Na avaliação da satisfação em relação ao uso do ambiente virtual, os residentes consideraram o *site* de fácil navegação (91%), adequado como ferramenta de aprendizagem (91%) e com *design* atrativo (79%). Eles relataram um conhecimento prévio ruim em reumatologia pediátrica (91%) e concordaram que houve um bom aprendizado por meio da metodologia (75%).

**Conclusões:**

O ambiente virtual de aprendizado em reumatologia pediátrica mostrou-se uma ferramenta de ensino eficaz e com altos índices de satisfação na sua utilização, fornecendo ao residente em pediatria um conhecimento adequado para avaliação e conduta inicial de pacientes com doenças reumáticas da infância.

## INTRODUCTION

Studies shows that the prevalence of pediatric rheumatic diseases ranges from 2,500 to 3,000 cases per million children and adolescents.^1^ According to the Brazilian Institute of Geography and Statistics (IBGE)’s 2017 projection, the Brazilian population below 19 years corresponds to 63 million children and adolescents, that is, probably up to 190 thousand Brazilian children and adolescents have a kind of rheumatic disease.^2^

Federal Council of Medicine’s data from 2017 shows only 77 registered pediatric rheumatologists nationwide, thus 1.2 pediatric rheumatologists for every million children and adolescents, well below the recommended by international pediatric rheumatology organizations, which is 2.5 professionals for one million children.^3,4^

A study conducted in Brazil in 2002 at eight pediatric rheumatology centers with juvenile idiopathic arthritis patients showed inadequate initial diagnosis in 84% of cases, leading to an average diagnostic delay of 1.4 years.^5^

The Brazilian Society of Pediatrics (SBP), through the basic curriculum for pediatrics recommended by the Global Pediatric Education Consortium (GPEC), recommends that, at the end of their training, the pediatric resident should be able to consider rheumatological disease, by choosing appropriate investigations, understanding the indication and complications of immunosuppressive treatment, and being able to orient families about treatment; and to recognize the suitable time to request an evaluation by a pediatric rheumatologist.^6^

There are no official data on the access of general pediatric residents in Brazil to pediatric rheumatology trainings. In Minas Gerais, for example, the unified selection process of 2017 offered 147 first-year pediatric vacancies in 30 medical residency programs, of which only four have pediatric rheumatology services at their teaching hospital. This estimate shows that possibly only 26% of Minas Gerais pediatric residents have any practical training in the field.

Data from 2010 in the United States showed that 34 to 40% of pediatric residency programs do not have pediatric rheumatology in their theoretical curriculum and between 50 and 70% do not have mandatory internship in the area.^7,8^

Since the 1960s, medical educators have started to develop computerized education systems.^9^ As for computer technologies for medical education, virtual learning environments (VLE) stand out as a safe and potential tool in the literature, supporting the teaching-learning process.^10^

In Canada, a survey was conducted with first-to-fourth-year pediatric residents to study the implementation of an online pediatric rheumatology teaching module, and 91% of respondents felt that, if available, an interactive online module would increase their learning in pediatric rheumatology.^11^

While there are not enough pediatric rheumatologists to train residents, better alternative training for pediatricians and pediatric residents are required, as these professionals are responsible for the first care of these children and this would impede delays in referral, diagnosis and treatment.

This study aimed to develop, apply and evaluate a teaching tool capable of spreading knowledge about pediatric rheumatology, providing pediatric residents with adequate knowledge for the initial evaluation and management of patients with childhood rheumatic diseases.

## METHOD

Case-control study aimed at the effectiveness and satisfaction of a VLE for pediatric rheumatology. The study was submitted to and approved by the local Research Ethics Committees.

Firstly, teachers of reference services in pediatric rheumatology (11 professionals) and pediatricians (four professionals) were invited to suggest ten topics on the subject that they considered of utmost practical significance in the context of pediatric residents’ training. After surveying all suggestions, a new survey was sent, to the same professionals through the Survey Monkey platform, so they could rate the themes suggested in the first request using a Likert scale from 1 to 7 (1 = strongly disagree and 7 = strongly agree). After statistical analysis, six themes were selected for the VLE modules.

The development technology used to build the web system by a specialized system analyst was ASP.NET 4 associated with the Bootstrap framework. The database used was Microsoft SQL Server 2012.

The main researcher in this project, trained and qualified in pediatric rheumatology, created video lessons and interactive clinical case sessions, which were then reviewed by the two co-authors, who are professors at a reference center of this specialty; scientific articles of free access were also selected to complement the teaching.

The virtual environment was tested by the researchers and consisted, in each module, of ten to 25 minutes of video lecture, an interactive case study and, on average, three suggested scientific articles for reading. The resident could only change modules after watching each video class in full and answering the entire interactive case report.

The 92 first- and second-year pediatric residents of two pediatric referral centers were emailed an invitation to participate in the Pediatric Rheumatology VLE. Residents of each institution were blind-randomized into case group and control group before their first access to the website.

During medical residency at both reference centers, residents perform an internship (20 hours of total workload) at the pediatric rheumatology outpatient clinic. At the outpatient clinic, they participate in the care of patients with rheumatological diseases, as well as in theoretical discussions about cases, receiving material for home study on the diseases handled.

In the first access, both residents of the case and the control group filled out a registration form with demographic data, signed the informed consent form and gave an electronic acceptance to participate in the project. They were assigned four months (from March 1, 2017 to June 30, 2017) to complete their participation in the project.

The control group responded to the pre-course test and, after four weeks, was invited to take the post-course test.

The case group, after answering the pre-course test, had access to VLE. At the end of the six learning modules, they answered the post-course test and filled a satisfaction questionnaire.

Both the pre-course and post-course tests consisted of 20 questions prepared by the authors about pediatric rheumatology on the subjects selected for VLE.

The literature lacks validated questionnaires to assess user satisfaction regarding VLEs in Portuguese. During the elaboration of the project, the VLE user satisfaction questionnaire validated in the master’s dissertation “Evaluation of the educational website in first aid” (“*Avaliação do website educacional em primeiros socorros*”)^12^ was found and used in this project, with the authorization of the thesis’ author. This questionnaire aims to evaluate the satisfaction of a VLE user regarding its overall appearance; ease of navigation and student attitude towards the website; adequacy of content and its apprehension. There is also an individual assessment of each theme in terms of readability, clarity and objectivity, photos, figures and animations, videos, audio and adequacy of each theme’s content.

To analyze the Likert scale for theme selection, the median values and standard deviations of the median were used. When, however, there was a tie, the values were selected according to quartiles.

To describe the database of the pre-course and post-course questionnaires for the case and control groups in relation to qualitative variables, absolute and relative frequencies were used, while in the description of quantitative variables we used measures of position, central tendency and dispersion. To assess the homogeneity between groups regarding the characterization variables, the Fisher’s exact test and the Chi-square test were used. In addition, the McNemar test was used to compare the clinical variables of the questionnaire related to the pre-and post-course. To verify the influence of time (pre-course and post-course) and group (case and control) on the mean percentage of correct answers of the 20 questions in both the pre-course and post-course tests, a linear regression model with interaction was used, adjusted for time and group, and then the appropriate contrasts were calculated. To describe the satisfaction bank, the percentages of satisfaction with the method were used. The Likert scale values ranging from 1 (strongly disagree/bad) to 3 (neither agree, disagree/good) were recoded to “disagree/dissatisfaction”, while values ranging from 4 (partly/agree) to 5 (strongly agree/excellent) were recoded as “agreement/satisfaction”. Statistical significance was considered at 5% and the software used in the analysis was R version 3.2.4.

## RESULTS

Fifteen physicians (11 specialists and 4 generalist pediatricians) suggested a total of 36 topics of pediatric rheumatology that they considered important for the learning of a pediatric resident in the first part of the research. Then, the same professionals classified the themes using a Likert scale from 1 to 7. After statistical analysis and elimination of similar themes, the following subjects were selected for the VLE:

1. History and physical examination in rheumatology.
2. Warning signs that may indicate rheumatic disease.
3. Musculoskeletal pain in childhood.
4. Differential diagnosis of acute arthritis.
5. Vasculitis: Kawasaki disease and Henoch-Schonlein purpura.
6. Juvenile idiopathic arthritis.

**Figure 1:**
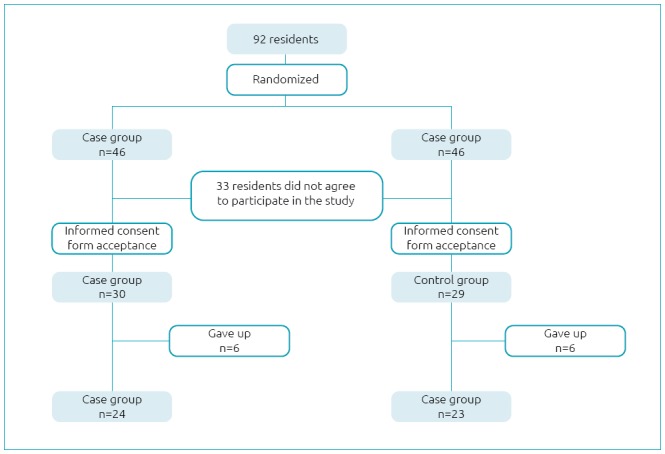
Flow chart of the sample of residents and losses throughout the project.

The participation of the groups in the project was completed by fulfilling the registration form and accepting the free informed consent form of 59 participants (64%); of these, 47 (51%) completed the participation in the study, divided between case and control groups (Figure 1).

In both study hospitals, over the two-year pediatric training, residents perform one-month outpatient internship in various specialties, including the pediatric rheumatology outpatient clinic, with total workload of approximately 20 hours. The variable “have you ever been to the pediatric rheumatology outpatient clinic?” was not homogeneous between the groups (p=0.011); in the case group, 20% of the individuals had done internship at the outpatient clinic, while in the control group 52% had. Statistical analysis using outpatient-controlled linear marginal regression showed no significant influence (p=0.583) of outpatient internship on the study outcome.

**Figure 2:**
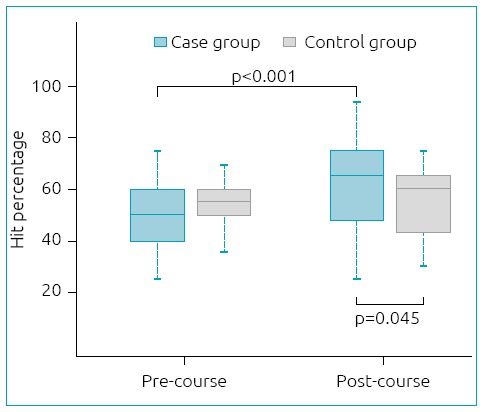
*Boxplot* of hit percentage in different times among groups.

Using linear marginal regression to evaluate the precourse and post-course questionnaires, the mean percentage of correct answers was 14% higher in post-course compared to pre-course (p<0.001). In the post-course test, there was a significant difference (p=0.045) between groups: the mean percentage of correct answers was 8% higher in the case group as opposed to the control group (Figure 2).

Table 1 shows the marginal linear regression controlled by the outpatient stage. This stage did not influence the results of comparison between tests, maintaining a statistically significant difference in the pre- and post-course tests for the case group (p<0.001), as well as in the post-course test comparison between both group (p=0.040).

**Table 1:** Comparison of the percentage of correct answers between times and between groups, controlled by internship at the pediatric rheumatology outpatient clinic.

Source		Descriptive analysis			Marginal linear regression		
		Mean	SD	β	SE (β)	95%CI	p-value
Case	Pre-course	50.0	13.5	-	-	-	-
	Post-course	63.1	17.8	14.0	3.5	7.2–20.8	<0.001
Control	Pre-course	55.0	9.5	-	-	-	-
	Post-course	55.4	12.9	1.5	2.3	-2.9–6.0	0.502
Pre-course	Control	55.0	9.5	-	-	-	-
	Case	50.0	13.5	-3.8	3.8	-11.1–3.6	0.316
Post-course	Control	55.4	12.9	-	-	-	-
	Case	63.1	17.8	8.7	4.2	0.4–16.9	0.040
Internship in outpatient clinics	No	54.6	14.5				
	Yes	57.4	13.5	1.8	3.3	-4.7–8.3	0.583

SD: standard deviation; EP: standard error; 95% CI: 95% confidence interval.

Case group residents spent on average 39 days (ranging from four hours to 120 days) to complete the VLE; and the control group showed a mean difference of 45 days between the pre-course and the post-course test (28 to 96 days), with no difference between groups (p=0.509).

Tables 2 and 3 list the result of the analysis of the satisfaction questionnaire on the website and the topics covered. It should be noted that users considered the website easy to navigate (92%), with well distributed information (83%), attractive (79%) and providing adequate learning (75%). Most residents (92%) agreed that the website suits well as a learning tool, that its objectives were clear at the outset, and that the understanding of its structure from the initial navigation is adequate. They reported poor prior knowledge about pediatric rheumatology (91%) and agreed that this methodology allowed good learning level (75%).

**Table 2:** Descriptive analysis of evaluation criteria of the virtual learning environment as for disagreement and agreement.

Variables		Disagreement/unsatisfaction		Agreement/satisfaction	
		n	%	n	n
Overall website design	1a - Website design is attractive and prompts the user to access the other pages.	5	20.8	19	79.2
	1b - Information is distributed logically and clearly, making it easy for students to find it.	4	16.7	20	83.3
Ease of navigation	2a - The organization of information is adequate.	2	8.3	22	91.7
	2b - The distribution of icons on screens is adequate.	1	4.2	23	95.8
	2c - Browsing the website page by page or from one link to another is appropriate.	8	33.3	16	66.7
Attitude towards the website	3a - Your willingness to use the website as a study tool was very good.	4	16.7	20	83.3
	3b - Your motivation for browsing the website was very good.	6	25	18	75.0
Adequacy of content and understanding	4a - Your prior knowledge of pediatric rheumatology before starting the discipline was very good.	22	91.7	2	8.3
	4b - Your learning in pediatric rheumatology at the end of the study through the website was adequate.	6	25	18	75.0
	4c - The website is suitable for use as a learning tool.	2	8.3	22	91.7
	4d - The website’s objectives were clear since the beginning.	2	8.3	22	91.7
	4e - Understanding the website’s structure from the initial navigation is adequate.	2	8.3	22	91.7
	4f - The division of content into themes favors learning in pediatric rheumatology.	-	-	24	100

**Table 3:** Descriptive analysis of criteria of the virtual learning environment themes as for disagreement and agreement.

Variables		Disagreement/unsatisfaction		Agreement/satisfaction	
		n	%	n	%
History and physical examination	Readability	1	4.2	23	95.8
	Clarity and objectivity	2	8.3	22	91.7
	Photos, figures and animations	6	25	18	75.0
	Videos	4	16.7	20	83.3
	Audio	7	29.2	17	70.8
	Content adequacy	2	8.3	22	91.7
Warning signs that may indicate rheumatic disease	Readability	-	-	24	100
	Clarity and objectivity	2	8.3	22	91.7
	Photos, figures and animations	7	29.2	17	70.8
	Videos	5	20.8	19	79.2
	Audio	7	29.2	17	70.8
	Content adequacy	2	8.3	22	91.7
Musculoskeletal pain in childhood	Readability	2	8.3	22	91.7
	Clarity and objectivity	2	8.3	22	91.7
	Photos, figures and animations	8	33.3	16	66.7
	Videos	4	16.7	20	83.3
	Audio	7	29.2	17	70.8
	Content adequacy	2	8.3	22	91.7
Differential diagnosis of acute arthritis	Readability	1	4.2	23	95.8
	Clarity and objectivity	2	8.3	22	91.7
	Photos, figures and animations	6	25	18	75.0
	Videos	5	20.8	19	79.2
	Audio	7	29.2	17	70.8
	Content adequacy	3	12.5	21	87.5
Juvenile Idiopathic Arthritis	Readability	-	-	24	100
	Clarity and objectivity	2	8.3	22	91.7
	Photos, figures and animations	6	25	18	75.0
	Videos	3	12.5	21	87.5
	Audio	5	20.8	19	79.2
	Content adequacy	2	8.3	22	91.7
Vasculitis in childhood: Kawasaki disease and Henoch Schonlein purpura	Readability	-	-	24	100
	Clarity and objectivity	1	4.2	23	95.8
	Photos, figures and animations	6	25	18	75.0
	Videos	2	8.3	22	91.7
	Audio	5	20.8	19	79.2
	Content adequacy	2	8.3	22	91.7

All topics had readability above 95.8%. The audio was the most criticized item, with satisfaction around 70% in all themes. In general, most participants were satisfied with the aspects evaluated in the questionnaire.

## DISCUSSION

In this pioneering, innovative study in our country, we observed that Pediatric Rheumatology VLE is an effective tool that brought a significant increase in knowledge between pre-course and post-course testing in the case group, which participated in the entire virtual environment (p<0.001), and with more effective learning compared to the control group (p=0.045). In addition, this tool was considered by 92% of users as suitable for learning.

The internet ushered in a new era that allowed students to quickly access information and virtual learning environments. However, not all educational sites are equally effective.^13^

Adult learning theories show that students retain more information when what is being taught is compatible with what they need to learn and that they need to be actively involved in the learning process.^14^

The SBP advocates the GPEC core pediatric curriculum, which recommends that, at the end of their training, the pediatric resident should be able to identify, evaluate, request appropriate propaedeutics and correctly refer the patient with rheumatic disease. Despite SBP’s recommendation, not all pediatric residency services have a pediatric rheumatology outpatient clinic.

Woodward & Harris’s work, published in 2013, aimed to understand the needs of American pediatricians and had about 40% of participants reporting that their pediatric training failed to recognize childhood rheumatic diseases, with 78% in favor of additional education methods in pediatric rheumatology.^15^ There are no relating data in our country, but the present study showed that in both centers, 91% of pediatric residents reported poor prior knowledge of pediatric rheumatology and agreed that the method allowed good learning (75%).

Our study had some limitations, such as poor adherence to the project, despite being compatible with the usual losses of online questionnaires.^16,17^ This may stem from the high workload of pediatric residents, with 60 hours of practical work per week, which makes free time to participate in distance learning initiatives short, allied to the study of other pediatric areas, resulting in limited time frame to participate in the project. We also observed that the frequency of participants who had already performed the internship at the pediatric rheumatology outpatient clinic was higher in the control group, which may represent a bias. Although this difference was not relevant in the statistical analysis of number of questions answered correctly, in relation to the absolute number of questions answered, the difference between the groups was 1.7 questions. Internship at the pediatric rheumatology outpatient clinic possibly influenced the performance for the good more than expected in the control group.

Activities aimed at training professionals to diagnose and, thus, promote early referral of suspected cases of pediatric rheumatology should be promoted by specialists in the field for an adequate care. Most stages in pediatric rheumatology outpatient clinics have a small workload in relation to the total time of pediatric residency. This results in less opportunity for pediatric residents to have contact with patients with the most prevalent diseases in the area. VLE is an attempt to supply this reality and may be complementary to traditional teaching activities, even in reference centers. Other efforts to train and update in pediatric rheumatology include SBP-sponsored guides, congresses and symposiums, as well as theoretical courses taught by specialists within their teaching units.

Our VLE can be included as an additional tool for this attempt, with the advantage of the online teaching format, which allows time flexibility for students and may also include access to information for other medical professionals such as pediatricians, ophthalmologists, orthopedists, dermatologists, and adult rheumatologists. VLE in pediatric rheumatology is useful for spreading knowledge in the area, increasing knowledge of pediatric residents, and improving assessment and initial management of patients with childhood rheumatic diseases.
